# The role of co-occurring insomnia and mental distress in the association between lumbar disc degeneration and low back pain related disability

**DOI:** 10.1186/s12891-023-06365-2

**Published:** 2023-04-14

**Authors:** Teija Mertimo, Eveliina Heikkala, Jaakko Niinimäki, Roberto Blanco Sequeiros, Juhani Määttä, Markku Kankaanpää, Petteri Oura, Jaro Karppinen

**Affiliations:** 1 Faculty of Medicine and Health Technology, Tampere University Hospital and University of Tampere, P.O. Box 607, Tampere, FI-33014 Finland; 2 Research Unit of Population Health, Faculty of Medicine, University of Oulu, P.O. Box 5000, Oulu, FI- 90014 Finland; 3 Medical Research Center Oulu, Oulu University Hospital and University of Oulu, P.O. Box 5000, Oulu, FI-90014 Finland; 4 Research Unit of Medical Imaging, Physics and Technology, Faculty of Medicine, University of Oulu, P.O. Box 5000, Oulu, FI-90014 Finland; 5 Department of Radiology, Turku University Hospital, Kiinamyllynkatu 4-8, Turku, FI-20520 Finland; 6 Research Unit of Health Sciences and Technology, Faculty of Medicine, University of Oulu, P.O. Box 5000, Oulu, FI-90014 Finland; 7 Department of Rehabilitation and Psychosocial Support, Tampere University Hospital, P.O. Box 2000, Tampere, FI-33521 Finland; 8 Rehabilitation Services of South Karelia Social and Health Care District, Valto Käkelän katu 3, Lappeenranta, FI- 53130 Finland; 9 Finnish Institute of Occupational Health, Aapistie 1, Oulu, FI-90220 Finland

**Keywords:** Insomnia, Lumbar disc degeneration, Low back pain, Magnetic resonance imaging, Mental distress, Pain-related disability, Prevalence, Finland, Northern Finland Birth Cohort 1966

## Abstract

**Background:**

Lumbar disc degeneration (LDD) is associated with low back pain (LBP). Although both insomnia and mental distress appear to influence the pain experience, their role in the association between LDD and LBP is uncertain. Our objective was to investigate the role of co-occurring insomnia and mental distress in the association between LDD and LBP-related disability.

**Methods:**

A total of 1080 individuals who had experienced LBP during the previous year underwent 1.5-T lumbar magnetic resonance imaging, responded to questionnaires, and participated in a clinical examination at the age of 47. Full data was available for 843 individuals. The presence of LBP and LBP-related disability (numerical rating scale, range 0–10) were assessed using a questionnaire. LDD was assessed by a Pfirrmann-based sum score (range 0–15, higher values indicating higher LDD). The role of insomnia (according to the five-item Athens Insomnia Scale) and mental distress (according to the Hopkins Symptom Check List-25) in the association between the LDD sum score and LBP-related disability was analyzed using linear regression with adjustments for sex, smoking, body mass index, education, leisure-time physical activity, occupational physical exposure, Modic changes, and disc herniations.

**Results:**

A positive association between LDD and LBP-related disability was observed among those with absence of both mental distress and insomnia (adjusted B = 0.132, 95% CI = 0.028–0.236, p = 0.013), and among those with either isolated mental distress (B = 0.345 CI = 0.039﻿–0.650, p = 0.028) or isolated insomnia (B = 0.207, CI = 0.040–0.373, p = 0.015). However, among individuals with co-occurring insomnia and mental distress, the association was not significant (B = -0.093, CI = -0.346-0.161, p = 0.470).

**Conclusions:**

LDD does not associate with LBP-related disability when insomnia and mental distress co-occur. This finding may be useful when planning treatment and rehabilitation that aim to reduce disability among individuals with LDD and LBP. Future prospective research is warranted.

**Supplementary Information:**

The online version contains supplementary material available at 10.1186/s12891-023-06365-2.

## Background

Low back pain (LBP) causes a significant burden to both individuals and societies worldwide [1]. It is especially consequential when it leads to disability [1]. LBP-related disability is highly prevalent, particularly in working-age populations [1]. For instance, LBP has been reported as the most common cause of sick leave and early retirement in Europe [2, 3]. Of individuals suffering from local LBP, only a small proportion has a specific pathological cause for the pain such as infection, fracture, or malignancy. In most cases, no pathological cause for pain can be identified [1, 4]. Lumbar disc degeneration (LDD) is repeatedly reported to have a significant association with LBP [5–10]. Still, it remains obscure why some studies [11, 12] have not observed such an association and whether underlying factors may influence the association between LDD and LBP. It is crucial that we gain more knowledge of the factors that influence the association between LDD and LBP-related disability to disentangle whether there are distinct subgroups of individuals among which LDD and LBP are differently associated.

Mental distress often co-occurs with insomnia [13] and it has been suggested that they both increase the risk of disabling LBP [1, 14]. For instance, mental distress has been associated with developing disability among individuals with LBP, while individuals with occasional LBP have been found to be at increased risk of developing persistent LBP with activity limitations when reporting insomnia [1, 14]. Insomnia, mental distress, and pain share a similar neurobiological background [15, 16]. It has been suggested that mental distress partially mediates the relationship between insomnia and pain [17]. However, the underlying factors and the direction of causality remain poorly understood [18].

Mental distress has been found to modify the association between LDD and LBP; if mental distress was not present, there was an association between LDD and LBP, but if mental distress was present, the association between LDD and LBP was lost [9]. However, in the previous study, the effect of insomnia was not disentangled from that of mental distress, even though the two often co-exist. The roles of isolated and co-occurring mental distress and insomnia in the association between LDD and LBP are poorly known. From a clinician’s perspective, and considering the burden of mental distress, insomnia, and LBP for individuals and society, it would be important to gain more knowledge on the interrelationships between these factors. It would also be important to disentangle the potential modifying roles of mental distress and insomnia in the association between LDD and LBP. This knowledge could be employed to achieve a treatment strategy that leads to a successful outcome.

## Methods

### Aim

The aim of this study was to investigate the potential role of co-occurring mental distress and insomnia (i.e., absence of both mental distress and insomnia, isolated mental distress, isolated insomnia, and co-occurring mental distress and insomnia) in the association between LDD and LBP-related disability using a large sample of Northern Finns reporting LBP. Based on our previous findings [9], we hypothesized that there is no significant relationship between LDD and LBP-related disability among people who have co-occurring mental distress and insomnia.

### Study Design

Cross-sectional study.

### Study sample

The Northern Finland Birth Cohort 1966 (NFBC1966) is a prospective longitudinal population-based cohort study (n = 12 058 live births) that initially comprised inhabitants of the two northernmost provinces of Finland (Oulu and Lapland). Pregnant women whose expected date of delivery was between January 1st and December 31st, 1966, were invited to the cohort. Cohort members have been followed since 1966 via, for example, regular postal questionnaires and clinical examinations (which comprised objective measurements of weight and height) [19, 20].

In this cross-sectional study, we used data (i.e., postal questionnaires, magnetic resonance imaging (MRI) scans, and clinical examination) from the most recent data collection point at the age of 47 in 2012–2014. A total of 7146 of the NFBC1966 cohort participants answered the questionnaires, 5832 participated in the clinical examinations, and 1540 underwent MRI of the lumbar spine. Of these 1540 participants, 1080 individuals had experienced LBP during the previous year prior to MRI, and were therefore included in the study. Those who had not had any LBP within this time period were excluded. The study sample used in the main analyses was based on 843 individuals who had the full data available.

### Assessment of lumbar MRI and evaluation of LDD

A total of 1540 NFBC1966 participants underwent MRI of the lumbar spine in 2012–2014. For the lumbar MRI we used 1.5-T equipment (Signa HDxt, General Electric, Milwaukee, WI) and T2-weighted fast-recovery fast spin-echo (frFSE) images in the sagittal and transverse planes, and T1-weighed fluid-attenuated inversion recovery sequence images in the sagittal plane. A detailed description of the MRI protocol has been presented in a previous publication [9]. The scans were assessed using NeaView Radiology software (Neagen Oy, Oulu, Finland), version 2.31.

A Pfirrmann-based LDD consensus reading was pursued as previously described [9]. In the first stage, MRI scans were independently assessed by two experienced musculoskeletal radiologists (J.N. and R.B.) and one highly experienced physiatrist with an extensive history in spinal imaging (J.K.). In the second stage, the first author (T.M.) pursued a consensus. The evaluators were blinded to all the other data and parameters used in the study [9]. The inter-rater reliability ranged from fair to good (κ = 0.39 to 0.79).

Based on the final LDD consensus, the overall burden of LDD was quantified by means of a Pfirrmann-based sum score variable by categorizing Grades I and II as 0, and Grades III, IV and V as 1, 2 and 3, respectively. The LDD sum score for five lumbar discs could thus theoretically range from 0 to 15, with higher values indicating higher LDD burden [9, 21].

### Assessment of low back pain

Data on LBP were collected using a questionnaire issued to the participants at the time of the MRI [9]. LBP was elicited using the following questions: 1) “Have you had any aches or pains in your lower back within the last 12 months? (no / yes)”. The anatomical area of LBP was illustrated by a drawing. If the response was positive, we asked them about pain-related disability at work, during leisure time and during sleep (altogether), rating it on a numerical rating scale (NRS) from 0 (no pain) to 10 (extremely bothersome pain/prevents activity/total disability).

### Assessment of mental distress and insomnia

As part of the questionnaires targeted to the cohort members, participants filled out the Hopkins Symptom Check List-25 (HSCL-25) [22, 23], and the five-item Athens Insomnia Scale (AIS-5) [24].

The HSCL-25 is a widely used instrument for screening symptoms of anxiety and depression [22, 23, 25, 26]. It consists of 25 questions, 10 on anxiety and 15 on depression [22]. The final score equals the average of the individual scores, ranging from 1.00 to 4.00^21^. In this study, the participants were divided into two categories depending on the presence of clinically relevant mental distress. The cut-off for clinically relevant mental distress in the HSCL-25 was set at 1.55 (< 1.55 no mental distress, ≥ 1.55 mental distress), as a cut-off of 1.55 is suggested by the previous literature [9, 23, 25–27]. It has been proposed that a cut-off of ≥ 1.55, defines a “probable psychiatric case” [25, 26].

The AIS-5 elicits the participants’ sleep problems over the past month [24, 28]. It has high reliability and validity in general and pain populations [24, 28]. It consists of four-point Likert scale (0–3) questions on sleep induction, nocturnal awakenings, morning awakenings, total sleep duration, and sleep quality [24]. In this study, the participants were divided into two categories depending on their summed score: no insomnia (< 4 points) and insomnia (≥ 4 points). The cut-off for insomnia in the AIS-5 was set at 4 in accordance with the previous literature [24, 28].

To study the influence of co-occurring insomnia and mental distress on the association between LDD and LBP-related disability, we formed a variable with the following four categories: (1) absence of both mental distress and insomnia, (2) isolated mental distress, (3) isolated insomnia, and (4) co-occurring mental distress and insomnia.

### Assessment of confounders

Based on previous studies, sex, smoking, body mass index (BMI), education, leisure-time physical activity, occupational physical exposure, and Modic changes and disc herniations presenting in lumbar MRI were considered potential confounders in the association between LDD and LBP [29–41]. The variables were recorded at the 47-year data collection point.

The participants’ weight and height were measured in the clinical examination by a trained nurse. BMI was calculated using the participant’s weight and height as kilograms per meter squared (kg/m^2^) and categorized according to the World Health Organization definition (normal weight: BMI < 25, overweight: BMI 25–30, and obesity: BMI > 30) [42].

Education level was elicited using the number of school years: < 9 school years, 9–12 school years, > 12 school years. This classification has also been used in a previous study [43] and is based on the Finnish education system.

Smoking was determined by two questions: (1) “Have you ever smoked cigarettes (yes/no)?” and (2) “Do you currently smoke (yes/no)?” Based on the answers, the participants were classified into three groups: (1) never-smokers, (2) former smokers and (3) current smokers [43].

To determine physical activity during leisure time, the participants were asked to estimate how often they take part in physical activity that causes at least some sweating and breathlessness (corresponding to moderate-to-vigorous intensity). The response alternatives were (1) daily, (2) 4–6 times a week, (3) 2–3 times a week, (4) once a week, (5) 2–3 times a month, and (6) once a month or less often. The participants were divided into three categories on the basis of their responses: “active” (at least four times a week), “moderately active” (1–3 times a week), and “inactive” (less than once a week) [34].

Occupational physical exposure was assessed as described previously [36]. Individuals were classified into two categories according to their occupational physical activity: “Low” (high-intensity tasks [i.e., hard physical labor, constant moving, and lifting heavy loads] performed rarely or occasionally) and “High” (at least one high-intensity task performed at least often).

The presence of lumbar disc herniations and Modic changes were also used as covariates. The protocols have been published previously [9, 43]. An experienced lumbar MRI reader (J.K.) evaluated the presence of disc herniations and dichotomized them as “no disc displacement or bulge”, or “ protrusion, extrusion or sequester” [43]. Modic changes were dichotomized as “present” or “absent” in accordance with previously published methodology [9, 43].

### Statistical analyses

Statistical analysis was performed using SPSS Statistics, version 27, 64-bit edition (IBM, Armonk, NY, USA). The threshold of statistical significance was set at P = 0.05. Descriptive statistics were used to present the distributions of LDD, LBP-related disability, and background variables in the pooled study sample and stratified by the co-occurring mental distress and insomnia categories; frequencies (n) and percentages (%) were used for categorical variables; and means with standard deviations (SD) or medians with interquartile ranges (IQR) were used for continuous variables. Differences between the co-occurring mental distress and insomnia categories were tested by Kruskal-Wallis and Chi-square tests; Bonferroni correction was applied in pairwise comparisons. Cronbach’s α was calculated as a measure of internal consistency for HSCL-25 and AIS-5.

The association between the LDD sum score (continuous predictor) and LBP-related disability (continuous outcome) was modeled using linear regression, with the beta coefficient (B), 95% confidence interval (CI) and P value. Both unadjusted and adjusted models were constructed. The final, adjusted, analyses were made on a complete-case basis.

To study the roles of mental distress and insomnia in the association between the LDD sum score and LBP-related disability, we stratified the regression models by the presence of mental distress and insomnia (the four-category variable). This approach was justified, as we observed a significant association between insomnia and LBP-related disability, and as we have also previously shown that mental distress modifies the association between LDD and LBP-related disability [9].

### Ethical approval

The study followed the principles of the Declaration of Helsinki and was approved by the Northern Ostrobothnia Hospital District Ethical Committee 94/2011 (12.12.2011). The NFBC1966 members took part voluntarily and signed their informed consent. All personal identity information was encrypted and pseudonymized before being granted to the researchers.

## Results

### Characteristics of the study population

The sample comprised 1080 individuals with LBP. Table 1 shows the characteristics of the present sample. Among the sample, the median LDD sum score was 4 (IQR 3–6), and the mean LBP-related disability was 4.7 (SD 2.5). Just over half were women and almost four-fifths were not obese. Over one-fifth of the sample reported mental distress and over a third had insomnia; both HSCL-25 and AIS-5 showed acceptable internal consistency (α = 0.924 and 0.801, respectively). Mental distress and insomnia co-existed among 12.2% of the participants, while nearly a half of the participants (49.3%) belonged to the absence of both mental distress and insomnia category (Table 1). A breakdown of characteristics by mental distress and insomnia category is presented in Table S1. The mean LBP -related disability was the highest in the co-occurring mental distress and insomnia category. Individuals with co-occurring mental distress and insomnia had lower education and leisure-time physical activity levels than individuals without mental distress or insomnia. There was no significant difference between the mental distress and insomnia groups in terms of sex distribution, BMI, smoking, occupational physical exposure, LDD sum score, and prevalence of Modic changes and disc herniations.

**Table 1:** Characteristics of study population (n = 1080) with low back pain

Variable	% (n)	Mean (SD) / Median (IQR)
Sex % (n)		
Men	44.9 (485)	
Women	54.9 (593)	
Missing	0.2 (2)	
Body Mass Index (kg/m^2^) % (n)		
< 25	38.8 (419)	
25–30	39.4 (426)	
> 30	21.3 (230)	
Missing	0.5 (5)	
Smoking % (n)		
Non-smoker	49.6 (536)	
Former	30.0 (324)	
Current	15.4 (166)	
Missing	5.0 (54)	
Education years % (n)		
< 9	3.6 (39)	
9–12	70.2 (758)	
> 12	22.9 (247)	
Missing	3.3 (36)	
Leisure-time physical activity (times/week) % (n)		
< 1	26.0 (281)	
1–3	54.6 (590)	
> 4	15.5 (167)	
Missing	3.9 (42)	
Occupational physical exposures % (n)		
Low	54.7 (591)	
High	38.6 (417)	
Missing	6.7 (72)	
LBP-related disability mean (SD)		4.7 (2.5)
Missing % (n)	5.5 (59)	
LDD sum score median (IQR)		4 (3–6)
Missing % (n)	3.2 (35)	
Modic changes % (n)		
Absent	27.9 (301)	
Present	68.6 (741)	
Missing	3.5 (38)	
Disc herniations % (n)		
No disc displacement or bulge	75.6 (816)	
Protrusion, extrusion or sequester	20.3 (219)	
Missing	4.2 (45)	
Mental distress % (n)		
No	75.6 (816)	
Yes	21.3 (230)	
Missing	3.1 (34)	
Insomnia % (n)		
No	57.3 (619)	
Yes	36.3 (392)	
Missing	6.4 (69)	
Mental distress and insomnia combined % (n)		
Absence of both mental distress and insomnia	49.3 (532)	
Isolated mental distress	7.9 (85)	
Isolated insomnia	24.1 (260)	
Co-occurring mental distress and insomnia	12.2 (132)	
Missing	6.6 (71)	

LBP, Low back pain; LDD, Lumbar disc degeneration; SD, standard deviation; IQR, interquartile ranges

**Table 2:** Association between lumbar disc degeneration (LDD) sum score and low back pain (LBP)-related disability, stratified by mental distress and insomnia

Stratification	Unadjusted B (95% CI) (n = 927*)	Adjusted^1^ B (95% CI) (n = 843**)
1. Absence of both mental distress and insomnia	**0.132 (0.044–0.221), p = 0.003** (n = 487)	**0.132 (0.028–0.236), p = 0.013** (n = 456)
2. Isolated mental distress	**0.236 (0.001–0.471), p = 0.049** (n = 75)	**0.345 (0.039–0.650), p = 0.028** (n = 68)
3. Isolated insomnia	**0.207 (0.068–0.346), p = 0.004** (n = 236)	**0.207 (0.040–0.373), p = 0.015** (n = 212)
4. Co-occurring mental distress and insomnia	-0.075 (-0.267-0.116), p = 0.438 (n = 129)	-0.093 (-0.346-0.161), p = 0.470 (n = 107)

^1: Adjusted for sex, smoking, body mass index, education, leisure-time physical activity, occupational physical exposure, Modic changes, and disc herniations^

^B, beta coefficients; CI, Confidence interval^

^Statistically significant values are bolded^

^*Data on LDD, LBP-related disability, and on both insomnia and mental distress available^

^**Full data available^

### The influence of co-occurring insomnia and mental distress on the association between LDD and LBP

Table 2 presents association between LDD sum score and LBP-related disability, stratified by mental distress and insomnia. We modeled the association between the LDD sum score and LBP-related disability among the participants who reported LBP in past 12 months, stratifying the models according to mental distress and insomnia (unadjusted n = 927, adjusted n = 843). A statistically significant association between LDD and LBP-related disability was found among those with absence of both mental distress and insomnia, isolated mental distress, and isolated insomnia. Among the participants reporting both mental distress and insomnia, we observed no statistically significant association between LDD and LBP-related disability. These findings were similar in both the unadjusted and adjusted models.

## Discussion

The aim of this study was to explore the role of co-occurring mental distress and insomnia in the association between LDD and LBP-related disability among a large sample of Northern Finns with LBP. A positive association between LDD and LBP-related disability was observed among those with absence of both mental distress and insomnia, and among those with either isolated mental distress or isolated insomnia. However, among individuals with co-occurring insomnia and mental distress, the association was not significant.

The prevalence of insomnia has been estimated to range between ∼5% and ∼15% [44–46] in the general population and between ∼30% and ∼50% among individuals with LBP [47–49]. In our study, the prevalence of insomnia among individuals with LBP during the previous year was 36.3%, which is of a similar magnitude to previous estimates [47–49]. The general prevalence of depression has been reported to be ∼5% [50–52] and the prevalence of mental distress among people with LBP ∼14% [49, 53]. In our study, the prevalence of mental distress was slightly higher, at ∼21%. The difference to the existing literature may be explained by our relatively low HSCL-25 cut-off [25]. Insomnia has been significantly associated with an increased risk of depression [54] and in present study 12.2% of the middle-aged individuals with LBP had both conditions. It seemed that LDD sum score median was similar among individuals with mental distress and insomnia than among the other participants, but they experienced LBP-related disability at a higher level.

Even though research activity around the predictors of LBP has been extensive, many questions remain unanswered [1]. Associations between LDD and LBP, as well as LBP and mental distress or insomnia have been suggested [1, 49, 55]. However, the factors influencing the association between LDD and LBP are poorly known, as we are aware of only one previous study [9] that has addressed this topic. The study found a significant association between LDD and LBP-related disability [9], and suggested that mental distress plays a modifying role in the association between LDD and LBP-related disability. A significant positive association was observed among individuals without mental distress according to HSCL-25 (< 1.55 points), the Beck Depression Inventory (< 13 points) or the Generalized Anxiety Disorder 7-item Scale (< 5 points), but not among individuals with mental distress [9]. The findings suggest that the LDD burden does not explain the LBP-related disability among individuals with mental distress. The need to identify individual LBP patients’ risk of co-occurring depression or insomnia has also been addressed or emphasized in other studies, [55, 56] but as far as we are aware, the recent study [9] is the first to explore the psychological factors that influence the association between LDD and LBP. The present study widens the knowledge in the field by disentangling the roles of isolated insomnia and co-occurring insomnia and mental distress. The present findings indicate that the association between LDD and LBP-related disability was lost among individuals with co-occurring mental distress and insomnia, concerning slightly over 10% of the participants. The results were independent of sex, smoking, BMI, education, leisure-time physical activity, occupational physical exposure, Modic changes, and disc herniations. Therefore, these factors are not expected to explain the non-significant association between LDD and LBP-related disability among participants with co-occurring mental distress and insomnia. Our outcome was specifically selected to capture LBP-related disability at work, during leisure time and during sleep.

Healthcare resources are limited and should be properly targeted. Although 85% of individuals with LBP are on sick leave for only a few days at most, the remaining 15% have longer sick leaves and account for half of the total number of sick leaves [3]. Data from a Norwegian population-based sample show that individuals with insomnia or mental distress have a lower rate of recovery from chronic LBP [57, 58]. Our findings thus underline the need to pay particular attention to potential presence of both insomnia and mental distress when treating patients with LBP in clinical work as these elements may be more important to LBP-related disability than LDD itself when both mental distress and insomnia are present. Additionally, to reduce the disability related to LBP, it may be beneficial to take co-occurring insomnia and mental distress into account in the treatment and rehabilitation of LBP. There are various treatment modalities for insomnia and depression among individuals with pain-related disability, for example cognitive behavioral therapy [59] and antidepressant medication [60]. Obviously, in addition to our study, further research with longitudinal data and prospective designs are urgently warranted.

### Strengths and weaknesses of the study

The strengths of this study were manifold. We used a general population sample of Northern Finns reporting LBP, and the sample size was relatively large. The assessments of insomnia and mental distress were based on reliable and validated questionnaires. LBP-related disability was chosen as the primary pain dimension, because it was perceived as a wide concept that captures pain-related disability at work, during leisure time, and during sleep. Adjustments were made for several potential confounders.

Our study also had limitations. As it had a cross-sectional design, neither causality nor the direction of associations could be addressed. The interrelationships between insomnia, pain and mental distress are potentially complex. Mental distress and insomnia were treated as dichotomous variables with two categories, which can be seen as a rough simplification of the underlying spectrum of symptoms. However, clinical use in Finland and previous studies [9, 23–28] have shown that these cut-offs effectively distinguish symptomatic individuals from asymptomatic ones.

## Conclusions

LDD does not seem to associate with the LBP-related disability when both insomnia and mental distress are present. A positive association between LDD and LBP-related disability was observed among those with absence of both mental distress and insomnia, and among those with either isolated mental distress or isolated insomnia. This finding may be useful when planning treatment and rehabilitation that aim to reduce LBP-related disability among individuals with LDD and LBP. In order to reduce the disability related to LDD, it may be beneficial to take co-occurring insomnia and mental distress into account in the treatment and rehabilitation of LBP. Further research with longitudinal data and prospective designs are urgently warranted.

## Electronic supplementary material

Below is the link to the electronic supplementary material.

Supplementary Material 1

## Data Availability

NFBC data are available from the University of Oulu, Infrastructure for Population Studies. Permission to use the data can be applied for research purposes via an electronic material request portal. In the use of data, we followed the EU general data protection regulation (679/2016) and the Finnish Data Protection Act. The use of personal data is based on a cohort participant’s written informed consent in their latest follow-up study, which may cause limitations to its use. Please contact the NFBC project centre (NFBCprojectcenter@oulu.fi) and visit the cohort website (www.oulu.fi/nfbc) for more information.

## List of abbreviations

AIS-5: the five-item Athens Insomnia Scale
B: Beta coefficient
BMI: Body mass index
CI: Confidence interval
HSCL-25: Hopkins Symptom Checklist-25
IQR: interquartile range
LBP: Low back pain
LDD: Lumbar disc degeneration
MRI: magnetic resonance imaging
NFBC1966: The Northern Finland Birth Cohort 1966
n: Number
NRS: Numerical rating scale
SD: standard deviation
