# Supplementary material for: The role of co-occurring insomnia and mental distress in the association between lumbar disc degeneration and low back pain related disability
Source: BMC Musculoskelet Disord. 2023 Apr 14;24:293. doi: 10.1186/s12891-023-06365-2 (PMC10103434; doi:10.1186/s12891-023-06365-2)
Supplement: Supplementary file 1 — Supplementary Material 1 [file 12891_2023_6365_MOESM1_ESM.docx]

| **Table S1.** Characteristics of study population with low back pain, stratified by the co-occurring mental distress and insomnia categories. | | | | | | | | | | |
| --- | --- | --- | --- | --- | --- | --- | --- | --- | --- | --- |
| Variable | 1) Absence of both mental distress and insomnia (n=532) | 2) Isolated mental distress (n=85) | 3) Isolated insomnia (n=260) | 4) Co-occurring mental distress and insomnia (n=132) | P values for pairwise comparisons after Bonferroni correctiont | | | | | |
|  |  |  |  |  | 1 vs 2 | 1 vs 3 | 1 vs 4 | 2 vs 3 | 2 vs 4 | 3 vs 4 |
| Sex % (n) ^a^ |  |  |  |  |  |  |  |  |  |  |
| Men | 47.4 (252) | 36.5 (31) | 46.5 (121) | 34.8 (46) | 0.366 | 1.000 | 0.072 | 0.594 | 1.000 | 0.174 |
| Women | 52.6 (280) | 63.5 (54) | 53.1 (138) | 64.4 (85) |  |  |  |  |  |  |
| *Missing* | 0 (0) | 0 (0) | 0.4 (1) | 0.8 (1) |  |  |  |  |  |  |
| Body Mass Index (kg/m^2^) % (n) ^a^ | |  |  |  |  |  |  |  |  |  |
| < 25 | 39.8 (212) | 49.4 (42) | 39.6 (103) | 29.5 (39) | 0.258 | 1.000 | 0.072 | 0.258 | 0.078 | 0.120 |
| 25–30 | 41.4 (220) | 27.1 (23) | 42.3 (110) | 40.2 (53) |  |  |  |  |  |  |
| > 30 | 18.8 (100) | 23.5 (20) | 18.1 (47) | 29.5 (39) |  |  |  |  |  |  |
| *Missing* | 0 (0) | 0 (0) | 0 (0) | 0.8 (1) |  |  |  |  |  |  |
| Smoking % (n) ^a^ |  |  |  |  |  |  |  |  |  |  |
| Non-smoker | 56.6 (301) | 41.2 (35) | 47.3 (123) | 43.9 (58) | 0.060 | 0.570 | 0.126 | 1.000 | 1.000 | 0.162 |
| Former | 28.6 (152) | 35.3 (30) | 34.2 (89) | 31.8 (42) |  |  |  |  |  |  |
| Current | 13.5 (72) | 23.5 (20) | 14.6 (38) | 21.2 (28) |  |  |  |  |  |  |
| *Missing* | 1.3 (7) | 0 (0) | 3.8 (10) | 3.0 (4) |  |  |  |  |  |  |
| Education years % (n) ^a^ | |  |  |  |  |  |  |  |  |  |
| < 9 | 2.1 (11) | 4.7 (4) | 3.8 (10) | 9.1 (12) | 0.288 | 0.876 | <0.001 | 1.000 | 1.000 | 0.288 |
| 9–12 | 70.7 (376) | 78.8 (67) | 72.7 (189) | 73.5 (97) |  |  |  |  |  |  |
| > 12 | 27.3 (145) | 16.5 (14) | 22.3 (58) | 15.9 (21) |  |  |  |  |  |  |
| *Missing* | 0 (0) | 0 (0) | 1.2 (3) | 1.5 (2) |  |  |  |  |  |  |
| Leisure-time physical activity (times/week) % (n) ^a^ | | | |  |  |  |  |  |  |  |
| < 1 | 21.8 (116) | 35.3 (30) | 26.9 (70) | 41.7 (55) | 0.084 | 1.000 | <0.001 | 1.000 | 0.984 | 0.030 |
| 1–3 | 61.1 (325) | 45.9 (39) | 55.0 (143) | 47.7 (63) |  |  |  |  |  |  |
| > 4 | 16.5 (88) | 17.6 (15) | 16.9 (44) | 9.1 (12) |  |  |  |  |  |  |
| *Missing* | 0.6 (3) | 1.2 (1) | 1.2 (3) | 1.5 (2) |  |  |  |  |  |  |
| Occupational physical exposures % (n) ^a^ | | |  |  |  |  |  |  |  |  |
| Low | 56.0 (298) | 56.5 (48) | 53.8 (140) | 59.1 (78) | 1.000 | 1.000 | 1.000 | 1.000 | 1.000 | 1.000 |
| High | 39.7 (211) | 35.3 (30) | 42.7 (111) | 35.6 (47) |  |  |  |  |  |  |
| *Missing* | 4.3 (23) | 8.2 (7) | 3.5 (9) | 5.3 (7) |  |  |  |  |  |  |
| LBP-related disability^b^ | |  |  |  |  |  |  |  |  |  |
| Mean (SD) | 4.4 (2.4) | 4.6 (2.6) | 4.9 (2.4) | 5.5 (2.5) | 1.000 | 0.024 | <0.001 | 1.000 | 0.174 | 0.270 |
| *Missing* % (n) | 6.0 (32) | 9.4 (8) | 5.4 (14) | 1.5 (2) |  |  |  |  |  |  |
| LDD sum score ^b^ | |  |  |  |  |  |  |  |  |  |
| Mean (SD) | 4 (3-6) | 4 (2-6) | 4 (3-6) | 4 (3-5) | 1.000 | 1.000 | 1.000 | 1.000 | 1.000 | 1.000 |
| *Missing* % (n) | 2.4 (13) | 2.4 (2) | 4.6 (12) | 0.8 (1) |  |  |  |  |  |  |
| Modic changes % (n) ^a^ | |  |  |  |  |  |  |  |  |  |
| Absent | 28.2 (150) | 30.6 (26) | 25.8 (67) | 28.8 (38) | 1.000 | 1.000 | 1.000 | 1.000 | 1.000 | 1.000 |
| Present | 69.4 (369) | 67.1 (57) | 70.0 (182) | 67.4 (89) |  |  |  |  |  |  |
| *Missing* | 2.4 (13) | 2.4 (2) | 4.2 (11) | 3.8 (5) |  |  |  |  |  |  |
| Disc herniations % (n) ^a^ | |  |  |  |  |  |  |  |  |  |
| No disc displacement or bulge | 77.1 (410) | 78.8 (67) | 73.1 (190) | 75.0 (99) | 1.000 | 1.000 | 1.000 | 1.000 | 1.000 | 1.000 |
| Protrusion, extrusion or sequester | 20.1 (107) | 18.8 (16) | 21.2 (55) | 20.5 (27) |  |  |  |  |  |  |
| *Missing* | 2.8 (15) | 2.4 (2) | 5.8 (15) | 4.5 (6) |  |  |  |  |  |  |

IQR, interquartile range; LBP, Low back pain; LDD, Lumbar disc degeneration; SD, standard deviation; vs, versus

Between-group differences analyzed using ^a^Chi squared test or ^b^Kruskal-Wallis test with Bonferroni correction for multiple testing
